# Mobile App-Based Interactive Care Plan for Migraine: Survey Study of Usability and Improvement Opportunities

**DOI:** 10.2196/66763

**Published:** 2025-03-26

**Authors:** Nathan P Young, Jennifer I Stern, Stephanie J Steel, Jon O Ebbert

**Affiliations:** 1 Department of Neurology, Mayo Clinic, 200 First St. SW Eighth Floor, Rochester, MN, 55905, United States, 1 5072842844; 2 Department of Internal Medicine, Mayo Clinic, Rochester, MN, United States

**Keywords:** migraine, remote monitoring, mobile app, mHealth, patient-reported outcomes, care plan, digital health, app, smartphone, eHealth, technology, survey study, headache, electronic health record, remote assessment, older adult, adult, electronic survey, pain, mobile phone, telehealth, telemedicine

## Abstract

**Background:**

We implemented a novel mobile app-based Migraine Interactive Care Plan (MICP) integrated with our electronic health records (EHRs). The MICP facilitates remote assessment of adult patients with migraine, educational content delivery, and care team communication. Feasibility of the MICP was demonstrated in a pilot implementation study.

**Objective:**

We aimed to assess the preferences and satisfaction of patients with migraine users of a mobile app-based care plan integrated with the EHR.

**Methods:**

An electronic survey was administered to a single cohort of MICP users between December 6, 2021, and December 30, 2021. The survey assessed patient preferences for which data to track, frequency of tracking, and satisfaction with the MICP. Survey responses were compared between subsets determined by patient-reported headache frequency and treatment with and without botulinum toxin and calcitonin gene-related peptide (CGRP) antagonist therapy. The Wilcoxon rank-sum test was used for continuous variables and the *χ*^2^ test or Fisher exact test for categorical variables.

**Results:**

The total sample size was 184 and the survey response rate was 30.4% (56/184). No significant differences in age (*P*=.26) or sex (*P*=.19) between respondents and nonrespondents were observed. Respondent median age was 42 (range 20‐72) years and 94.6% (53/56) were female. Headache frequency was (1) 0 to 8 days (26/56, 46.4%), (2) 9 to 14 days (12/56, 21.4%), and (3) 15 or more days (18/56, 32.1%). No difference was observed in any survey responses based on headache frequency or treatment. The majority of respondents preferred to track headache days weekly (30/56, 53.6%) or daily (15/56, 26.8%) and preferred to change the frequency of headache tracking reminders (42/56, 75%). Respondents were somewhat or very interested in daily tracking personal observations in free text (41/52, 78.8%), medication treatment (43/52, 82.7%) and treatment response (39/56, 69.6%), class of medication treatment (36/52, 69.2%), severity of functional impairment (39/56, 69.6%), type of functional impairment (35/53, 66%), headache day (40/54, 74.1%), and headache pain level on a scale of 1 to 10 (38/53, 71.7%). Respondents agreed or strongly agreed that the education content was useful (31/51, 60.8%) but lacked personalization (25/51, 49%). Most respondents agreed or strongly agreed that they were satisfied with the MICP (38/50, 76%) and that it helped them communicate with their care team (38/53, 71.7%).

**Conclusions:**

Most MICP users were motivated to track headache frequency, medication treatment with response, functional impairment, and pain intensity. Opportunities to improve the MICP include (1) allowing patients to change the frequency of assessments and notifications; (2) recording personal observations or comments through free text, which may include headache triggers; (3) assessment of headache severity using a 1 to 10 pain scale; and (4) tailoring headache education based on frequency and severity (episodic vs chronic migraine). These observations may be useful to improve the usability of the MICP and similar EHR-integrated migraine care platforms that others may develop.

## Introduction

### Background

Patients with migraine often face significant barriers in access to evidence-based care, with approximately one-quarter of those with episodic migraine and less than 5% of those with chronic migraine receiving appropriate treatment [1-4]. Access to care is especially challenging for women, racial and ethnic minority groups, and patients living in rural areas [5]. Novel health care delivery models may improve care access when resources are limited and projected to worsen [2,6]. Studies have demonstrated that telemedicine improves access to migraine care, and that it is associated with favorable outcomes including patient and provider satisfaction [7,8]. Internet or smartphone app-based remote assessment and monitoring of migraine [9,10] may increase the efficiency of care delivery and facilitate telemedicine, electronic [11], and face-to-face visits while delivering migraine educational content. Headache clinicians are comfortable treating patients through telemedicine and most have indicated a high level of interest in prescribing migraine apps [12] while citing the importance of integrating remote monitoring data into the electronic health record (EHR) [13]. Smartphone-based apps may also improve diagnosis [14], deliver educational content [15], guide behavioral treatment [16-18], and advance clinical trials [19]. There are improvement opportunities with respect to accessibility of apps for headache [20].

### Previous Work

We implemented a novel smartphone app-based Migraine Interactive Care Plan (MICP) integrated with the EHR (Epic, Epic Systems) in our community neurology practice, and potentially available to other institutions using Epic EHR. Development and testing of the MICP has been previously published [15]. Feasibility of the MICP was demonstrated in a pilot implementation study, but usability did not reach a predetermined threshold of 75% of users completing at least one electronically assigned task 127/171 (74.3%) [15].

Access to the MICP was ordered by a provider at the time of a face-to-face neurology consult and integrated into the existing EHR app (ie, Epic MyChart platform). This integration allowed patient access to their medical record, care team messaging, and appointment management. The MICP was designed to facilitate remote monitoring of adult patients with migraine, deliver educational content, and streamline care delivery. Key features of the MICP included (1) weekly assessments of headache days, treatment days, reduced function, and absenteeism; (2) monthly assessments of patient satisfaction (Likert scale) with their migraine treatment plan; (3) Migraine Disability Assessment (MIDAS) survey [21] assessment every 3 months; (4) scheduled delivery of migraine education content; and (5) weekly assessment of medication compliance and concerns and electronic messaging with the care team if needed.

### Goal of This Study

In this study, we aimed to identify opportunities to improve MICP usability by surveying MICP users to assess their satisfaction and preferences and whether they differ based on migraine frequency or type of preventive treatment.

## Methods

### Ethical Considerations

This quality improvement project was granted an exemption waiver for written consent from the Mayo Clinic (IRB 20‐000606). All respondents electronically signed the Health Insurance Portability and Accountability Act (HIPAA) agreement before starting the survey. No compensation was offered to complete the survey. All survey responses were deidentified before analysis, and the data were on a secure server.

### Overview

A novel electronic survey was developed and formatted (on Qualtrics). Between December 6, 2021, and December 30, 2021, all MICP users (n=184) who completed MICP enrollment and completed at least one task received an email invitation to a secure web-based survey with a personalized letter from a physician on the migraine care team (NPY) explaining the goals of the survey and encouragement to voluntarily participate. The survey cohort had all completed a neurological headache consultation within a general community neurology practice before enrollment in the MICP. Weekly email reminders were sent by a study coordinator to nonrespondents for a total of 4 weeks. Only 1 survey completion was allowed. Demographic variables, including age and sex, were retrieved from the EHR for respondents and nonrespondents and were compared using the *t* test. Categorical variables were summarized. The overall survey response rate was defined by the percentage of surveys returned. The analysis of the results from each individual question included only the completed response to the question and nonresponses to individual questions were excluded. Survey responses were compared between subsets determined by patient reported headache frequency (0 to 8 days, 9 to 14 days, or 15 or more days), survey self-reported treatment with and without botulinum toxin and calcitonin gene-related peptide (CGRP) antagonist therapy. The Wilcoxon rank-sum test was used for continuous variables and *χ*^2^ test or Fisher exact test for categorical variables using a 2-tailed α level of 5%. Missing survey response data were excluded from analysis. Analysis was performed using SAS software (version 9.4; SAS Inc).

## Results

The survey response rate was 30.4% (56/184). No significant differences in age (*P*=.26) or sex (*P*=.19) between respondents and nonrespondents were observed. Table 1 summarizes the survey responses of all respondents, and a comprehensive table including the subgroup analysis and *P* values are summarized in Multimedia Appendix 1 and Multimedia Appendix 2.

**Table 1.** Survey responses of migraine users of the Migraine Interactive Care Plan.

Survey question	Total respondents (N=56), n (%)
How often would you prefer to track your headache days on a smartphone app?	
Daily	15 (26.8)
Weekly	30 (53.6)
Monthly	8 (14.3)
Every 3 months	3 (5.4)
How often would you like to be reminded to record a headache day on a smartphone app?	
Daily	14 (25)
Weekly	33 (58.9)
Monthly	7 (12.5)
Every 3 months	2 (3.6)
Would you prefer to control and change the frequency of headache tracking reminders?	
Yes	42 (75)
No	14 (25)
In addition to headache days, please tell us what else you are interested in tracking with your headache days?	
Migraine/headache triggers	41 (73.2)
Stress level	38 (67.8)
Sleep	37 (66.1)
Step count	9 (16.1)
Heart rate	4 (7.1)
Diet	16 (28.6)
Exercise	15 (26.8)
Diet calories	9 (16.1)
Please rate your level of interest and motivation to track, on a daily basis, the following factors in the Mayo Clinic Migraine Care Plan	
Headache days (yes/no)	
Not at all interested	3 (5.6)
Not very interested	3 (5.6)
Neutral	8 (14.8)
Somewhat interested	15 (27.8)
Very Interested	25 (46.3)
If yes, then pain level on scale 1‐10	
Not at all interested	3 (5.7)
Not very interested	1 (1.9)
Neutral	11 (20.8)
Somewhat interested	17 (32.1)
Very Interested	21 (39.6)
Functional impairment (mild, moderate, or severe)	
Not at all interested	3 (5.7)
Not very interested	2 (3.8)
Neutral	9 (17)
Somewhat interested	17 (32.1)
Very Interested	22 (41.5)
What type of functions was impaired (work, school, family, or personal)	
Not at all interested	3 (5.7)
Not very interested	4 (7.5)
Neutral	11 (20.8)
Somewhat interested	16 (30.2)
Very Interested	19 (35.8)
Did you take medication (yes or no)	
Not at all interested	3 (5.8)
Not very interested	0 (0)
Neutral	6 (11.5)
Somewhat interested	14 (26.9)
Very Interested	29 (55.8)
If yes, then which type of medication (pick from multiple in list)	
Not at all interested	5 (9.6)
Not very interested	1 (1.9)
Neutral	10 (19.2)
Somewhat interested	19 (36.5)
Very interested	17 (32.7)
Response to medication	
Not at all interested	3 (5.8)
Not very interested	1 (1.9)
Neutral	9 (17.3)
Somewhat interested	15 (28.8)
Very interested	24 (46.2)
Your own personal observations or comments (free text)	
Not at all interested	3 (5.8)
Not very interested	1 (1.9)
Neutral	7 (13.5)
Somewhat interested	20 (38.5)
Very interested	21 (40.4)
Please rate your level of agreement with the following statements about the Mayo Clinic Migraine Care Plan.	
I felt confident using the Mayo Clinic Care Plan	
Strongly disagree	1 (1.9)
Disagree	2 (3.8)
Neither agree nor disagree	8 (15.1)
Agree	24 (45.3)
Strongly agree	18 (34)
The Mayo Clinic Care Plan app was easy to use	
Strongly disagree	2 (3.8)
Disagree	4 (7.5)
Neither agree nor disagree	11 (20.8)
Agree	21 (39.6)
Strongly agree	15 (28.3)
The equipment helped in my care at home	
Strongly disagree	1 (1.9)
Disagree	8 (15.1)
Neither agree nor disagree	23 (43.4)
Agree	11 (20.8)
Strongly agree	10 (18.9)
I felt comfortable interacting with my care team through the Mayo Clinic Care Plan	
Strongly disagree	0 (0)
Disagree	3 (5.8)
Neither agree nor disagree	7 (13.5)
Agree	24 (46.2)
Strongly agree	18 (34.6)
It helped me better understand my condition	
Strongly disagree	1 (1.9)
Disagree	7 (13.2)
Neither agree nor disagree	23 (43.4)
Agree	12 (22.6)
Strongly agree	10 (18.9)
It helped me understand how to care for myself	
Strongly disagree	1 (1.9)
Disagree	9 (17)
Neither agree nor disagree	24 (45.3)
Agree	9 (17)
Strongly agree	10 (18.9)
It helped me understand what I should be tracking throughout my care	
Strongly disagree	1 (1.9)
Disagree	5 (9.4)
Neither agree nor disagree	24 (45.3)
Agree	14 (26.4)
Strongly agree	9 (17)
It helped me understand what steps I could take to improve my health	
Strongly disagree	1 (1.9)
Disagree	8 (15.1)
Neither agree nor disagree	25 (47.2)
Agree	11 (20.8)
Strongly agree	8 (15.1)
It helped me communicate with my care team	
Strongly disagree	2 (3.8)
Disagree	5 (9.4)
Neither agree nor disagree	8 (15.1)
Agree	27 (50.9)
Strongly agree	11 (20.8)
It helped to inform me when to contact my care team about concerning symptoms	
Strongly disagree	2 (3.8)
Disagree	9 (17)
Neither agree nor disagree	15 (28.3)
Agree	17 (32.1)
Strongly agree	10 (18.9)
Please rate your level of agreement with the following statements about the education provided while using the Mayo Clinic Care Plan.	
The educational materials were useful to me	
Strongly disagree	2 (3.9)
Disagree	6 (11.8)
Neither agree nor disagree	12 (23.5)
Agree	22 (43.1)
Strongly agree	9 (17.6)
The information was easy to understand	
Strongly disagree	1 (2)
Disagree	0 (0)
Neither agree nor disagree	11 (21.6)
Agree	26 (51)
Strongly agree	13 (25.5)
I was comfortable with how often I received educational materials	
Strongly disagree	2 (3.9)
Disagree	3 (5.9)
Neither agree nor disagree	11 (21.6)
Agree	26 (51)
Strongly agree	9 (17.6)
I was able to find the educational materials when I needed them	
Strongly disagree	3 (5.9)
Disagree	2 (3.9)
Neither agree nor disagree	13 (25.5)
Agree	25 (49)
Strongly agree	8 (15.7)
The educational materials matched my personal needs	
Strongly disagree	1 (2)
Disagree	5 (9.8)
Neither agree nor disagree	20 (39.2)
Agree	19 (37.3)
Strongly agree	6 (11.8)
The educational information from the Mayo Clinic Care Plan matched the information received from my Mayo Clinic Care Team	
Strongly disagree	1 (2)
Disagree	0 (0)
Neither agree nor disagree	18 (35.3)
Agree	21 (41.2)
Strongly agree	11 (21.6)
Please rate your level of agreement with the following statements.	
I would recommend the Mayo Clinic Care Plan to others with similar health condition(s)	
Strongly disagree	1 (2)
Disagree	2 (4)
Neither agree nor disagree	8 (16)
Agree	24 (48)
Strongly agree	15 (30)
Overall, I am satisfied with the Mayo Clinic Care Plan	
Strongly disagree	1 (2)
Disagree	4 (8)
Neither agree nor disagree	7 (14)
Agree	25 (50)
Strongly agree	13 (26)

The median age of respondents was 42 (range 20‐72) years and 94.6% (53/56) were female. Respondents reported treatment with an injectable CGRP antagonist (17/56, 30.4%) or botulinum toxin (22/56, 39.3%). Patient-reported headache frequency at the time of the survey was (1) 0 to 8 days (26/56, 46.4%), (2) 9 to 14 days (12/56, 21.4%), and (3) 15 or more days (18/56, 32.1%). No differences in survey responses were observed comparing patients reporting CGRP antagonist treatment versus no CGRP treatment, botulinum toxin treatment versus no botulinum toxin treatment, or between headache frequency groups at the time of the survey.

In terms of frequency, respondents had the highest preference for tracking headache days weekly (30/56, 53.6%) followed by daily (15/56, 26.8%) and monthly (8/56, 14.3%), with a tracking frequency of every 3 months being the least preferred (3/56, 5.4%). Respondents indicated a similar preference for how frequently they would like to be reminded to record a headache day. Most respondents preferred to have control over the frequency of headache tracking reminders (42/56, 75%). In addition to tracking headache days, most respondents indicated a preference for tracking migraine and headache triggers (42/56, 73.2%), stress level (38/56, 67.8%), and sleep (37/56, 66.1%). Fewer participants preferred tracking diet (16/56, 28.6%), exercise (15/56, 26.8%), caloric intake (9/56, 16.1%), step count (9/56, 16.1%), and heart rate (4/56, 7.1%).

When asked about the level of interest and motivation to track on a daily basis, respondents were somewhat or very interested in tracking personal observations or comments in free text (41/52, 78.8%), medication treatment (43/52, 82.7%), response to medication (39/56, 69.6%), class of medication treatment chosen from a list (36/52, 69.2%), functional impairment graded mild, moderate, or severe (39/56, 69.6%), type of function impaired including work, school, family, personal (35/53, 66%), headache day (40/54, 74.1%), and headache pain level on scale 1‐10 (38/53, 71.7%).

The majority of respondents agreed or strongly agreed with statements about the Migraine Care Plan that they “felt confident using” (42/53, 79.2%), “was easy to use” (36/53, 68%), “felt comfortable interacting with my care team” (42/52, 80.8%), “helped to the inform me when to contact my care team about concerning symptoms” (27/53, 50.9%), and “helped me communicate with my care team” (38/53, 71.7%). A minority of respondents agreed or strongly agreed with statements that “the equipment helped in my care at home” (21/53, 40%), “helped me better understand my condition” (22/53, 41.5%), “understand how to care for myself” (19/53, 35.8%), “understand what I should be tracking throughout my care” (23/53, 43.4%), and “understand steps I can take to improve my health” (19/53, 35.8%).

Respondents agreed or strongly agreed that the education materials were useful (31/51, 60.8%) and easy to understand (39/51, 76.5%), but fewer agreed that the education content “matched my personal needs” (25/51, 49%). Most respondents agreed or strongly agreed that they were satisfied with the Migraine Care Plan (38/50, 76%).

## Discussion

### Principal Findings

We assessed the satisfaction and preferences of MICP and did not observe differences based on migraine frequency or type of preventive treatment. However, analysis of all respondents highlight that the most notable MICP improvement opportunities include allowing patients to enter free text observational data and adjust the frequency of monitoring.

In addition, we observed that most respondents prefer to track headache outcomes that are of interest to clinicians engaged in migraine management, including headache days, treatment days and response, and functional impairment [22]. Most respondents in this study preferred weekly tracking as well as the option to change the frequency of tracking reminders along with the ability to enter free text data and observations and rate the severity of pain on a 1 to 10 pain scale, all of which were not included in the MICP. Most respondents were not interested in monitoring non-headache data such as diet, exercise, caloric intake, or heart rate. Most respondents agreed that the educational content delivered was useful but that the content lacked personalization. Most respondents were satisfied with the MICP and felt that it helped them communicate with their care team, supporting ongoing use in our practice.

### Key MICP Improvement Opportunities and Comparison With Previous Work

#### Free Text Capability

We observed that 78.8% (41/52) of respondents were somewhat or very interested in recording their own free text observations. This capability was not included in the MICP because of concern that free text data might be difficult for clinicians to interpret or might not be clinically actionable or easy to summarize in the EHR. Minen et al [23] also reported a similar preference of a patient with migraine for recording free text data. Although such data may be difficult for clinicians to efficiently review within the EHR and contain clinical irrelevant observations [22], it may be important for patient engagement and improve usability. As we enter the era of artificial intelligence [24], such free text data [25] may be a powerful data set for artificial intelligence natural language processing tools that may be able to quickly summary such data or use in algorithm development to facilitate patient assessment and management [26-29].

#### Frequency of Monitoring

Only 15/56 (26.8%) respondents in our study indicated a preference for daily monitoring, and only 5/171 (2.9%) users in the pilot study of the MICP responded to nearly all assigned assessment tasks [15]. Raffaeli et al described a cohort of highly engaged users of a commercial app that engaged with the app daily for more than 7‐13 months. This cohort consisted of only 1.8% of 85,000 active app users who used the app daily for 7 months [10]. These observations suggest that sustained engagement on a daily or weekly basis may not be a realistic goal for most patients. Whether giving patients the option to change the frequency of assessments would improve overall engagement is not known.

Our MICP allowed weekly tracking of the number of headache days, treatment days, and days with functional impairment. However, most of our patients indicated an interest in changing this tracking frequency (42/56, 75%). The range of different preferences in tracking frequency supports allowing patients to choose the frequency of monitoring even though the accuracy of the data may be less precise the longer a patient may be asked to recall. It is also possible that the preference for tracking frequency might depend on migraine frequency, for example, patients with less frequent episodic migraine may prefer less frequent monitoring than a patient with uncontrolled chronic migraine. We suspect that as patients are effectively treated with appropriate acute and preventative migraine therapies, headache frequency and disability decrease such that daily and even weekly monitoring would no longer be necessary. A study in a larger population of patients is needed to determine if flexibility would increase overall engagement.

Patient preferences for tracking frequency may not always align with clinician preference or insurance requirements. In addition, different tracking frequencies serve different purposes. For example, the diagnosis of episodic versus chronic migraine depends on 3 months of data, whereas a shorter period may be ideal for making changes in medications or other aspects of care.

The survey suggests that patients would prefer to control their tracking and tracking reminder frequency. Individualization of tracking and reminder frequency may enhance patient engagement and understanding, especially for those with a new diagnosis of migraine. Requiring monthly assessments of headache days, treatment days, function, and satisfaction will allow the clinician access to data that will inform best migraine care. For example, monthly tracking may quickly uncover medication overuse behavior. It may also allow the clinician early detection of the transition between episodic and chronic migraine, which in turn may allow new treatment options such as onabotulinumtoxin A. The MIDAS assessment every 3 months may not be needed except for research purposes to allow comparison with other studies or migraine care model intervention. Monthly data trends would be simple to summarize and easy to interpret for busy clinicians.

#### Educational Materials

Respondents agreed or strongly agreed that the education materials were useful (31/51, 60.8%) but the education did not always match personal needs (25/51, 49%). The migraine educational content was generally applicable and may be useful to a patient with a new migraine diagnosis. Most MICP users in this study had frequent migraine that led to neurology consultation, and as most were treated with botox or CGRP, it can be assumed majority failed to respond to multiple first-line preventive options. Education specific to chronic migraine was minimal. The MICP may be improved by delivering educational content that is tailored to the migraine diagnosis or current treatment. Delivering content that is already known or simply not of interest may disengage patients.

### Strengths

We present survey findings from a unique group of patients that used a novel digital tool directly interfacing with the EHR within a community neurology practice. We assessed preferences for a broad range of features that may both inform migraine care and promote patient engagement. We compared respondents with nonrespondents and between subsets of patients. The low response rate was similar to other survey studies of the same population [6,30,31].

### Limitations

Limitations include the small sample size, and the overall survey response rate was low, reducing the reproducibility and generalizability of our findings. In addition, the study was underpowered to detect differences in user preferences based on headache frequency or treatment type. Most patients in the survey likely had chronic migraine or frequent episodic migraine as is typical of our specialty practice. The majority of patients were treated with botulinum toxin injections or CGRP antagonist therapy at the time of the study, and the majority of patients reported less than 15 headache days per month, likely reflecting the effectiveness of their current treatment plan. We did not retrospectively review records to confirm the migraine diagnosis documented in the medication record. In addition, most of the population we assessed were white and female, with high migraine frequency and access to a neurology specialty clinic and for these reasons our observations are not generalizable to other patient populations or health care systems. Finally, we did not reassess the frequency or level of engagement of MICP users who completed the survey, increasing potential response bias.

### Conclusions

Most MICP users were motivated to track clinically actionable data, including headache frequency, medication treatment with response, functional impairment, and pain intensity; features that should be maintained in the MICP and similar tools that others may develop. Opportunities to improve the MICP that may be useful for other similar remote monitoring tools include allowing patients to (1) change the frequency of assessments and notifications; (2) record personal observations or comments through free text, which may include headache triggers, accompanying symptoms, and prodrome and postdrome symptoms; (3) report headache severity using a 1 to 10 pain scale; and (4) tailor headache education based on frequency and severity (episodic vs chronic migraine). To balance the needs for patient engagement with the changes above, the MICP may be simplified by requiring a monthly assessment of headache days, treatment days, function, and satisfaction with treatment plan without MIDAS every 3 months. Remote assessment and monitoring of patients with migraine is feasible, and continued work to refine remote monitoring tools informed by patient preferences and reassessment of user preferences within diverse and larger populations, including both episodic and chronic patients with migraine, is needed.

## Supplementary material

10.2196/66763 Multimedia Appendix 1 Survey responses in subsets based on headache frequency.

10.2196/66763 Multimedia Appendix 2 Survey responses in subsets based on migraine treatment type.

## Abbreviations

CGRP: calcitonin gene-related peptide
EHR: electronic health record
MICP: Migraine Interactive Care Plan
MIDAS: Migraine Disability Assessment
