# Supplementary material for: Mobile App-Based Interactive Care Plan for Migraine: Survey Study of Usability and Improvement Opportunities
Source: JMIR Form Res. 2025 Mar 26;9:e66763. doi: 10.2196/66763 (PMC11964953; doi:10.2196/66763)
Supplement: Multimedia Appendix 1 [file formative-v9-e66763-s001.docx]

**Multimedia Appendix 1. Survey Responses in Subsets Based on Headache Frequency**

| **Survey Question** | **Headache days 0 to 8**  **(N=26)** | **Headache days 9 to 14**  **(N=12)** | **Headache days ≥15**  **(N=18)** | **Headache days**  ***P* value** |
| --- | --- | --- | --- | --- |
| **How often would you prefer to track your headache days on a smartphone application?** |  |  |  | .8282 |
| Daily | 6 (23.1%) | 5 (41.7%) | 4 (22.2%) |  |
| Weekly | 15 (57.7%) | 5 (41.7%) | 10 (55.6%) |  |
| Monthly | 3 (11.5%) | 2 (16.7%) | 3 (16.7%) |  |
| Every 3 months | 2 (7.7%) | 0 (0%) | 1 (5.6%) |  |
| **How often would you like to be reminded to record a headache day on a smartphone application?** |  |  |  | .5847 |
| Daily | 5 (19.2%) | 5 (41.7%) | 4 (22.2%) |  |
| Weekly | 16 (61.5%) | 6 (50%) | 11 (61.1%) |  |
| Monthly | 3 (11.5%) | 1 (8.3%) | 3 (16.7%) |  |
| Every 3 months | 2 (7.7%) | 0 (0%) | 0 (0%) |  |
| **Would you prefer to control and change the frequency of headache tracking reminders?** |  |  |  | .3145 |
| Yes | 18 (69.2%) | 11 (91.7%) | 13 (72.2%) |  |
| No | 8 (30.8%) | 1 (8.3%) | 5 (27.8%) |  |
| **In addition to headache days, please tell us what else you are interested in tracking with your headache days?** |  |  |  |  |
| **Migraine/headache triggers** |  |  |  | 1.0000 |
| Yes | 21 (80.8%) | 11 (91.7%) | 9 (50%) |  |
| **Stress Level** |  |  |  | 1.0000 |
| Yes | 18 (69.2%) | 9 (75%) | 11 (61.1%) |  |
| **Sleep** |  |  |  | 1.0000 |
| Yes | 20 (76.9%) | 7 (58.3%) | 10 (55.6%) |  |
| **Step Count** |  |  |  | 1.0000 |
| Yes | 4 (15.4%) | 3 (25%) | 2 (11.1%) |  |
| **Heart Rate** |  |  |  | 1.0000 |
| Yes | 1 (3.8%) | 1 (8.3%) | 2 (11.1%) |  |
| **Diet** |  |  |  | 1.0000 |
| Yes | 8 (30.8%) | 5 (41.7%) | 3 (16.7%) |  |
| **Exercise** |  |  |  | 1.0000 |
| Yes | 8 (30.8%) | 2 (16.7%) | 5 (27.8%) |  |
| **Diet calories** |  |  |  | 1.0000 |
| Yes | 5 (19.2%) | 2 (16.7%) | 2 (11.1%) |  |
| **Please rate your level of interest and motivation to track, on a daily basis, the following factors in the Mayo Clinic Migraine Care Plan** |  |  |  |  |
| **Headache days (yes/no)** |  |  |  | .5929 |
| Not at all interested | 2 (8.3%) | 0 (0%) | 1 (5.6%) |  |
| Not very interested | 2 (8.3%) | 1 (8.3%) | 0 (0%) |  |
| Neutral | 5 (20.8%) | 2 (16.7%) | 1 (5.6%) |  |
| Somewhat interested | 7 (29.2%) | 2 (16.7%) | 6 (33.3%) |  |
| Very Interested | 8 (33.3%) | 7 (58.3%) | 10 (55.6%) |  |
| **If yes, then pain level on scale 1- 10** |  |  |  | .2466 |
| Not at all interested | 2 (8.7%) | 0 (0%) | 1 (5.6%) |  |
| Not very interested | 0 (0%) | 1 (8.3%) | 0 (0%) |  |
| Neutral | 6 (26.1%) | 3 (25%) | 2 (11.1%) |  |
| Somewhat interested | 8 (34.8%) | 1 (8.3%) | 8 (44.4%) |  |
| Very Interested | 7 (30.4%) | 7 (58.3%) | 7 (38.9%) |  |
| **Functional impairment (mild, moderate, severe)** |  |  |  | .6846 |
| Not at all interested | 2 (8.7%) | 0 (0%) | 1 (5.6%) |  |
| Not very interested | 1 (4.3%) | 1 (8.3%) | 0 (0%) |  |
| Neutral | 6 (26.1%) | 1 (8.3%) | 2 (11.1%) |  |
| Somewhat interested | 7 (30.4%) | 4 (33.3%) | 6 (33.3%) |  |
| Very Interested | 7 (30.4%) | 6 (50%) | 9 (50%) |  |
| **What type of functions was impaired (work, school, family, personal)** |  |  |  | .3199 |
| Not at all interested | 2 (8.7%) | 0 (0%) | 1 (5.6%) |  |
| Not very interested | 1 (4.3%) | 2 (16.7%) | 1 (5.6%) |  |
| Neutral | 7 (30.4%) | 3 (25%) | 1 (5.6%) |  |
| Somewhat interested | 8 (34.8%) | 2 (16.7%) | 6 (33.3%) |  |
| Very Interested | 5 (21.7%) | 5 (41.7%) | 9 (50%) |  |
| **Did you take medication (yes/no)** |  |  |  | .3128 |
| Not at all interested | 2 (8.7%) | 0 (0%) | 1 (5.9%) |  |
| Not very interested | 0 (0%) | 0 (0%) | 0 (0%) |  |
| Neutral | 4 (17.4%) | 2 (16.7%) | 0 (0%) |  |
| Somewhat interested | 8 (34.8%) | 2 (16.7%) | 4 (23.5%) |  |
| Very Interested | 9 (39.1%) | 8 (66.7%) | 12 (70.6%) |  |
| **If yes, then which class (s) of medication (pick from multiple in list)** |  |  |  | .2512 |
| Not at all interested | 3 (13%) | 0 (0%) | 2 (11.8%) |  |
| Not very interested | 1 (4.3%) | 0 (0%) | 0 (0%) |  |
| Neutral | 4 (17.4%) | 5 (41.7%) | 1 (5.9%) |  |
| Somewhat interested | 10 (43.5%) | 3 (25%) | 6 (35.3%) |  |
| Very interested | 5 (21.7%) | 4 (33.3%) | 8 (47.1%) |  |
| **Response to medication** |  |  |  | .6103 |
| Not at all interested | 2 (8.7%) | 0 (0%) | 1 (5.9%) |  |
| Not very interested | 1 (4.3%) | 0 (0%) | 0 (0%) |  |
| Neutral | 5 (21.7%) | 3 (25%) | 1 (5.9%) |  |
| Somewhat interested | 7 (30.4%) | 2 (16.7%) | 6 (35.3%) |  |
| Very interested | 8 (34.8%) | 7 (58.3%) | 9 (52.9%) |  |
| **Your own personal observations/comments (free text)** |  |  |  | .7842 |
| Not at all interested | 2 (8.7%) | 0 (0%) | 1 (5.9%) |  |
| Not very interested | 0 (0%) | 0 (0%) | 1 (5.9%) |  |
| Neutral | 4 (17.4%) | 2 (16.7%) | 1 (5.9%) |  |
| Somewhat interested | 9 (39.1%) | 4 (33.3%) | 7 (41.2%) |  |
| Very interested | 8 (34.8%) | 6 (50%) | 7 (41.2%) |  |
| **Please rate your level of agreement with the following statements about the Mayo Clinic Migraine Care Plan.** |  |  |  |  |
| **I felt confident using the Mayo Clinic Care Plan** |  |  |  | .2335 |
| Strongly disagree | 0 (0%) | 0 (0%) | 1 (5.9%) |  |
| Disagree | 1 (4.2%) | 0 (0%) | 1 (5.9%) |  |
| Neither agree nor disagree | 3 (12.5%) | 4 (33.3%) | 1 (5.9%) |  |
| Agree | 13 (54.2%) | 2 (16.7%) | 9 (52.9%) |  |
| Strongly agree | 7 (29.2%) | 6 (50%) | 5 (29.4%) |  |
| **The Mayo Clinic Care Plan app was easy to use** |  |  |  | .8635 |
| Strongly disagree | 1 (4.2%) | 0 (0%) | 1 (5.9%) |  |
| Disagree | 2 (8.3%) | 0 (0%) | 2 (11.8%) |  |
| Neither agree nor disagree | 5 (20.8%) | 4 (33.3%) | 2 (11.8%) |  |
| Agree | 10 (41.7%) | 4 (33.3%) | 7 (41.2%) |  |
| Strongly agree | 6 (25%) | 4 (33.3%) | 5 (29.4%) |  |
| **The equipment helped in my care at home** |  |  |  | .4047 |
| Strongly disagree | 0 (0%) | 0 (0%) | 1 (5.9%) |  |
| Disagree | 2 (8.3%) | 2 (16.7%) | 4 (23.5%) |  |
| Neither agree nor disagree | 14 (58.3%) | 3 (25%) | 6 (35.3%) |  |
| Agree | 4 (16.7%) | 3 (25%) | 4 (23.5%) |  |
| Strongly agree | 4 (16.7%) | 4 (33.3%) | 2 (11.8%) |  |
| **I felt comfortable interacting with my care team through the Mayo Clinic Care Plan** |  |  |  | 0.3584 |
| Strongly disagree |  |  |  |  |
| Disagree | 0 (0%) | 1 (8.3%) | 2 (11.8%) |  |
| Neither agree nor disagree | 1 (4.3%) | 2 (16.7%) | 4 (23.5%) |  |
| Agree | 13 (56.5%) | 5 (41.7%) | 6 (35.3%) |  |
| Strongly agree | 9 (39.1%) | 4 (33.3%) | 5 (29.4%) |  |
| **It helped me better understand my condition** |  |  |  | 0.0494 |
| Strongly disagree | 0 (0%) | 0 (0%) | 1 (5.9%) |  |
| Disagree | 0 (0%) | 5 (41.7%) | 2 (11.8%) |  |
| Neither agree nor disagree | 11 (45.8%) | 4 (33.3%) | 8 (47.1%) |  |
| Agree | 7 (29.2%) | 1 (8.3%) | 4 (23.5%) |  |
| Strongly agree | 6 (25%) | 2 (16.7%) | 2 (11.8%) |  |
| **It helped me understand how to care for myself** |  |  |  | 0.2038 |
| Strongly disagree | 0 (0%) | 0 (0%) | 1 (5.9%) |  |
| Disagree | 1 (4.2%) | 5 (41.7%) | 3 (17.6%) |  |
| Neither agree nor disagree | 12 (50%) | 4 (33.3%) | 8 (47.1%) |  |
| Agree | 5 (20.8%) | 1 (8.3%) | 3 (17.6%) |  |
| Strongly agree | 6 (25%) | 2 (16.7%) | 2 (11.8%) |  |
| **It helped me understand what I should be tracking throughout my care** |  |  |  | 0.5769 |
| Strongly disagree | 0 (0%) | 0 (0%) | 1 (5.9%) |  |
| Disagree | 1 (4.2%) | 2 (16.7%) | 2 (11.8%) |  |
| Neither agree nor disagree | 10 (41.7%) | 7 (58.3%) | 7 (41.2%) |  |
| Agree | 8 (33.3%) | 1 (8.3%) | 5 (29.4%) |  |
| Strongly agree | 5 (20.8%) | 2 (16.7%) | 2 (11.8%) |  |
| **It helped me understand what steps I could take to improve my health** |  |  |  | 0.6467 |
| Strongly disagree | 0 (0%) | 0 (0%) | 1 (5.9%) |  |
| Disagree | 2 (8.3%) | 3 (25%) | 3 (17.6%) |  |
| Neither agree nor disagree | 14 (58.3%) | 5 (41.7%) | 6 (35.3%) |  |
| Agree | 4 (16.7%) | 2 (16.7%) | 5 (29.4%) |  |
| Strongly agree | 4 (16.7%) | 2 (16.7%) | 2 (11.8%) |  |
| **It helped me communicate with my care team** |  |  |  | 0.9004 |
| Strongly disagree | 0 (0%) | 1 (8.3%) | 1 (5.9%) |  |
| Disagree | 2 (8.3%) | 1 (8.3%) | 2 (11.8%) |  |
| Neither agree nor disagree | 4 (16.7%) | 2 (16.7%) | 2 (11.8%) |  |
| Agree | 12 (50%) | 5 (41.7%) | 10 (58.8%) |  |
| Strongly agree | 6 (25%) | 3 (25%) | 2 (11.8%) |  |
| **It helped to inform me when to contact my care team about concerning symptoms** |  |  |  | 0.5830 |
| Strongly disagree | 0 (0%) | 1 (8.3%) | 1 (5.9%) |  |
| Disagree | 3 (12.5%) | 4 (33.3%) | 2 (11.8%) |  |
| Neither agree nor disagree | 7 (29.2%) | 3 (25%) | 5 (29.4%) |  |
| Agree | 8 (33.3%) | 2 (16.7%) | 7 (41.2%) |  |
| Strongly agree | 6 (25%) | 2 (16.7%) | 2 (11.8%) |  |
| **Please rate your level of agreement with the following statements about the education provided while using the Mayo Clinic Care Plan.** |  |  |  |  |
| **The educational materials were useful to me** |  |  |  | 0.3760 |
| Strongly disagree | 0 (0%) | 0 (0%) | 2 (11.8%) |  |
| Disagree | 2 (9.1%) | 3 (25%) | 1 (5.9%) |  |
| Neither agree nor disagree | 4 (18.2%) | 4 (33.3%) | 4 (23.5%) |  |
| Agree | 11 (50%) | 4 (33.3%) | 7 (41.2%) |  |
| Strongly agree | 5 (22.7%) | 1 (8.3%) | 3 (17.6%) |  |
|  |  |  |  |  |
| **The information was easy to understand** |  |  |  | 0.7149 |
| Strongly disagree | 0 (0%) | 0 (0%) | 1 (5.9%) |  |
| Disagree |  |  |  |  |
| Neither agree nor disagree | 4 (18.2%) | 4 (33.3%) | 3 (17.6%) |  |
| Agree | 13 (59.1%) | 5 (41.7%) | 8 (47.1%) |  |
| Strongly agree | 5 (22.7%) | 3 (25%) | 5 (29.4%) |  |
| **I was comfortable with how often I received educational materials** |  |  |  | 0.4073 |
| Strongly disagree | 0 (0%) | 1 (8.3%) | 1 (5.9%) |  |
| Disagree | 2 (9.1%) | 0 (0%) | 1 (5.9%) |  |
| Neither agree nor disagree | 2 (9.1%) | 5 (41.7%) | 4 (23.5%) |  |
| Agree | 13 (59.1%) | 4 (33.3%) | 9 (52.9%) |  |
| Strongly agree | 5 (22.7%) | 2 (16.7%) | 2 (11.8%) |  |
| **I was able to find the educational materials when I needed them** |  |  |  | 0.7314 |
| Strongly disagree | 0 (0%) | 1 (8.3%) | 2 (11.8%) |  |
| Disagree | 1 (4.5%) | 1 (8.3%) | 0 (0%) |  |
| Neither agree nor disagree | 7 (31.8%) | 3 (25%) | 3 (17.6%) |  |
| Agree | 10 (45.5%) | 5 (41.7%) | 10 (58.8%) |  |
| Strongly agree | 4 (18.2%) | 2 (16.7%) | 2 (11.8%) |  |
| **The educational materials matched my personal needs** |  |  |  | 0.1014 |
| Strongly disagree | 0 (0%) | 0 (0%) | 1 (5.9%) |  |
| Disagree | 0 (0%) | 3 (25%) | 2 (11.8%) |  |
| Neither agree nor disagree | 9 (40.9%) | 7 (58.3%) | 4 (23.5%) |  |
| Agree | 9 (40.9%) | 2 (16.7%) | 8 (47.1%) |  |
| Strongly agree | 4 (18.2%) | 0 (0%) | 2 (11.8%) |  |
| **The educational information from the Mayo Clinic Care Plan matched the information received from my Mayo Clinic Care Team** |  |  |  | 0.6522 |
| Strongly disagree | 0 (0%) | 0 (0%) | 1 (5.9%) |  |
| Disagree |  |  |  |  |
| Neither agree nor disagree | 7 (31.8%) | 6 (50%) | 5 (29.4%) |  |
| Agree | 10 (45.5%) | 3 (25%) | 8 (47.1%) |  |
| Strongly agree | 5 (22.7%) | 3 (25%) | 3 (17.6%) |  |
| **Please rate your level of agreement with the following statements.** |  |  |  |  |
| **I would recommend the Mayo Clinic Care Plan to others with similar health condition(s)** |  |  |  | 0.6740 |
| Strongly disagree | 0 (0%) | 0 (0%) | 1 (5.9%) |  |
| Disagree | 0 (0%) | 1 (8.3%) | 1 (5.9%) |  |
| Neither agree nor disagree | 4 (19%) | 1 (8.3%) | 3 (17.6%) |  |
| Agree | 10 (47.6%) | 5 (41.7%) | 9 (52.9%) |  |
| Strongly agree | 7 (33.3%) | 5 (41.7%) | 3 (17.6%) |  |
| **Overall, I am satisfied with the Mayo Clinic Care Plan** |  |  |  | 0.8679 |
| Strongly disagree | 0 (0%) | 0 (0%) | 1 (5.9%) |  |
| Disagree | 1 (4.8%) | 2 (16.7%) | 1 (5.9%) |  |
| Neither agree nor disagree | 3 (14.3%) | 2 (16.7%) | 2 (11.8%) |  |
| Agree | 11 (52.4%) | 5 (41.7%) | 9 (52.9%) |  |
| Strongly agree | 6 (28.6%) | 3 (25%) | 4 (23.5%) |  |
