# Supplementary material for: Mobile App-Based Interactive Care Plan for Migraine: Survey Study of Usability and Improvement Opportunities
Source: JMIR Form Res. 2025 Mar 26;9:e66763. doi: 10.2196/66763 (PMC11964953; doi:10.2196/66763)
Supplement: Multimedia Appendix 2 [file formative-v9-e66763-s002.docx]

**Multimedia Appendix 2: Survey Responses in Subsets Based on Migraine Treatment Type**

| **Survey Question** | **CGRP treatment**  **(N=17)** | **No-CGRP treatment**  **(N=39)** | **CGRP treatment**  ***P* value** | **b. toxin treatment**  **(N=22)** | **No- b. toxin treatment**  **(N=33)** | **b. toxin treatment**    ***P* value** |
| --- | --- | --- | --- | --- | --- | --- |
| **How often would you prefer to track your headache days on a smartphone application?** |  |  | .5790 |  |  | .5790 |
| Daily | 8 (36.4%) | 7 (21.2%) |  | 8 (36.4%) | 7 (21.2%) |  |
| Weekly | 11 (50%) | 18 (54.5%) |  | 11 (50%) | 18 (54.5%) |  |
| Monthly | 2 (9.1%) | 6 (18.2%) |  | 2 (9.1%) | 6 (18.2%) |  |
| Every 3 months | 1 (4.5%) | 2 (6.1%) |  | 1 (4.5%) | 2 (6.1%) |  |
| **How often would you like to be reminded to record a headache day on a smartphone application?** |  |  | .2868 |  |  | .4574 |
| Daily | 3 (17.6%) | 11 (28.2%) |  | 8 (36.4%) | 6 (18.2%) |  |
| Weekly | 10 (58.8%) | 23 (59%) |  | 11 (50%) | 21 (63.6%) |  |
| Monthly | 4 (23.5%) | 3 (7.7%) |  | 2 (9.1%) | 5 (15.2%) |  |
| Every 3 months | 0 (0%) | 2 (5.1%) |  | 1 (4.5%) | 1 (3%) |  |
| **Would you prefer to control and change the frequency of headache tracking reminders?** |  |  | .2402 |  |  | .0697 |
| Yes | 11 (64.7%) | 31 (79.5%) |  | 14 (63.6%) | 28 (84.8%) |  |
| No | 6 (35.3%) | 8 (20.5%) |  | 8 (36.4%) | 5 (15.2%) |  |
| **In addition to headache days, please tell us what else you are interested in tracking with your headache days?** |  |  |  |  |  |  |
| **Migraine/headache triggers** |  |  | 1.0000 |  |  | 1.0000 |
| Yes | 14 (82.4%) | 27 (69.2%) |  | 15 (68.2%) | 25 (75.8%) |  |
| **Stress Level** |  |  | 1.0000 |  |  | 1.0000 |
| Yes | 11 (64.7%) | 27 (69.2%) |  | 14 (63.6%) | 23 (69.7%) |  |
| **Sleep** |  |  | 1.0000 |  |  | 1.0000 |
| Yes | 12 (70.6%) | 25 (64.1%) |  | 14 (63.6%) | 22 (66.7%) |  |
| **Step Count** |  |  | 1.0000 |  |  | 1.0000 |
| Yes | 3 (17.6%) | 6 (15.4%) |  | 3 (13.6%) | 6 (18.2%) |  |
| **Heart Rate** |  |  | 1.0000 |  |  | 1.0000 |
| Yes | 1 (5.9%) | 3 (7.7%) |  | 1 (4.5%) | 3 (9.1%) |  |
| **Diet** |  |  | 1.0000 |  |  | 1.0000 |
| Yes | 4 (23.5%) | 12 (30.8%) |  | 6 (27.3%) | 10 (30.3%) |  |
| **Exercise** |  |  | 1.0000 |  |  | 1.0000 |
| Yes | 4 (23.5%) | 11 (28.2%) |  | 7 (31.8%) | 8 (24.2%) |  |
| **Diet calories** |  |  | . |  |  | 1.0000 |
| Yes | 5 (29.4%) | 4 (10.3%) |  | 3 (13.6%) | 6 (18.2%) |  |
| **Please rate your level of interest and motivation to track, on a daily basis, the following factors in the Mayo Clinic Migraine Care Plan** |  |  |  |  |  |  |
| **Headache days (yes/no)** |  |  | 0.9370 |  |  | .1488 |
| Not at all interested | 1 (5.9%) | 2 (5.4%) |  | 2 (9.1%) | 1 (3.2%) |  |
| Not very interested | 1 (5.9%) | 2 (5.4%) |  | 1 (4.5%) | 2 (6.5%) |  |
| Neutral | 2 (11.8%) | 6 (16.2%) |  | 1 (4.5%) | 7 (22.6%) |  |
| Somewhat interested | 6 (35.3%) | 9 (24.3%) |  | 4 (18.2%) | 10 (32.3%) |  |
| Very Interested | 7 (41.2%) | 18 (48.6%) |  | 14 (63.6%) | 11 (35.5%) |  |
| **If yes, then pain level on scale 1- 10** |  |  | .4197 |  |  | .1896 |
| Not at all interested | 1 (6.3%) | 2 (5.4%) |  | 2 (9.1%) | 1 (3.3%) |  |
| Not very interested | 0 (0%) | 1 (2.7%) |  | 0 (0%) | 1 (3.3%) |  |
| Neutral | 2 (12.5%) | 9 (24.3%) |  | 2 (9.1%) | 9 (30%) |  |
| Somewhat interested | 8 (50%) | 9 (24.3%) |  | 6 (27.3%) | 10 (33.3%) |  |
| Very Interested | 5 (31.3%) | 16 (43.2%) |  | 12 (54.5%) | 9 (30%) |  |
| **Functional impairment (mild, moderate, severe)** |  |  | .8463 |  |  | .3258 |
| Not at all interested | 1 (6.3%) | 2 (5.4%) |  | 2 (9.1%) | 1 (3.3%) |  |
| Not very interested | 0 (0%) | 2 (5.4%) |  | 0 (0%) | 2 (6.7%) |  |
| Neutral | 2 (12.5%) | 7 (18.9%) |  | 3 (13.6%) | 6 (20%) |  |
| Somewhat interested | 6 (37.5%) | 11 (29.7%) |  | 5 (22.7%) | 11 (36.7%) |  |
| Very Interested | 7 (43.8%) | 15 (40.5%) |  | 12 (54.5%) | 10 (33.3%) |  |
| **What type of functions was impaired (work, school, family, personal)** |  |  | .9573 |  |  | .8101 |
| Not at all interested | 1 (6.3%) | 2 (5.4%) |  | 2 (9.1%) | 1 (3.3%) |  |
| Not very interested | 1 (6.3%) | 3 (8.1%) |  | 1 (4.5%) | 3 (10%) |  |
| Neutral | 3 (18.8%) | 8 (21.6%) |  | 4 (18.2%) | 6 (20%) |  |
| Somewhat interested | 6 (37.5%) | 10 (27%) |  | 6 (27.3%) | 10 (33.3%) |  |
| Very Interested | 5 (31.3%) | 14 (37.8%) |  | 9 (40.9%) | 10 (33.3%) |  |
| **Did you take medication (yes/no)** |  |  | .5488 |  |  | .7752 |
| Not at all interested | 1 (6.7%) | 2 (5.4%) |  | 2 (9.1%) | 1 (3.4%) |  |
| Not very interested | 1 (6.7%) | 5 (13.5%) |  | 3 (13.6%) | 3 (10.3%) |  |
| Neutral | 6 (40%) | 8 (21.6%) |  | 0 (0%) | 0 (0%) |  |
| Somewhat interested | 7 (46.7%) | 22 (59.5%) |  | 5 (22.7%) | 9 (31%) |  |
| Very Interested | 0 (0%) | 0 (0%) |  | 12 (54.5%) | 16 (55.2%) |  |
| **If yes, then which class (s) of medication (pick from multiple in list)** |  |  | .2554 |  |  | .8057 |
| Not at all interested | 2 (13.3%) | 3 (8.1%) |  | 3 (13.6%) | 2 (6.9%) |  |
| Not very interested | 1 (6.7%) | 0 (0%) |  | 0 (0%) | 1 (3.4%) |  |
| Neutral | 1 (6.7%) | 9 (24.3%) |  | 4 (18.2%) | 6 (20.7%) |  |
| Somewhat interested | 7 (46.7%) | 12 (32.4%) |  | 7 (31.8%) | 11 (37.9%) |  |
| Very interested | 4 (26.7%) | 13 (35.1%) |  | 8 (36.4%) | 9 (31%) |  |
| **Response to medication** |  |  | .1387 |  |  | .6968 |
| Not at all interested | 1 (6.7%) | 2 (5.4%) |  | 2 (9.1%) | 1 (3.4%) |  |
| Not very interested | 1 (6.7%) | 0 (0%) |  | 0 (0%) | 1 (3.4%) |  |
| Neutral | 1 (6.7%) | 8 (21.6%) |  | 5 (22.7%) | 4 (13.8%) |  |
| Somewhat interested | 7 (46.7%) | 8 (21.6%) |  | 6 (27.3%) | 9 (31%) |  |
| Very interested | 5 (33.3%) | 19 (51.4%) |  | 9 (40.9%) | 14 (48.3%) |  |
| **Your own personal observations/comments (free text)** |  |  | .6216 |  |  | .5643 |
| Not at all interested | 1 (6.7%) | 2 (5.4%) |  | 2 (9.1%) | 1 (3.4%) |  |
| Not very interested | 0 (0%) | 1 (2.7%) |  | 1 (4.5%) | 0 (0%) |  |
| Neutral | 2 (13.3%) | 5 (13.5%) |  | 3 (13.6%) | 3 (10.3%) |  |
| Somewhat interested | 8 (53.3%) | 12 (32.4%) |  | 9 (40.9%) | 11 (37.9%) |  |
| Very interested | 4 (26.7%) | 17 (45.9%) |  | 7 (31.8%) | 14 (48.3%) |  |
| **Please rate your level of agreement with the following statements about the Mayo Clinic Migraine Care Plan.** |  |  |  |  |  |  |
| **I felt confident using the Mayo Clinic Care Plan** |  |  | .8444 |  |  | .6967 |
| Strongly disagree | 0 (0%) | 1 (2.7%) |  | 0 (0%) | 1 (3.3%) |  |
| Disagree | 1 (6.3%) | 1 (2.7%) |  | 1 (4.5%) | 1 (3.3%) |  |
| Neither agree nor disagree | 3 (18.8%) | 5 (13.5%) |  | 2 (9.1%) | 6 (20%) |  |
| Agree | 6 (37.5%) | 18 (48.6%) |  | 10 (45.5%) | 13 (43.3%) |  |
| Strongly agree | 6 (37.5%) | 12 (32.4%) |  | 9 (40.9%) | 9 (30%) |  |
| **The Mayo Clinic Care Plan app was easy to use** |  |  | .5092 |  |  | .2372 |
| Strongly disagree | 0 (0%) | 2 (5.4%) |  | 0 (0%) | 2 (6.7%) |  |
| Disagree | 1 (6.3%) | 3 (8.1%) |  | 2 (9.1%) | 2 (6.7%) |  |
| Neither agree nor disagree | 3 (18.8%) | 8 (21.6%) |  | 2 (9.1%) | 8 (26.7%) |  |
| Agree | 9 (56.3%) | 12 (32.4%) |  | 9 (40.9%) | 12 (40%) |  |
| Strongly agree | 3 (18.8%) | 12 (32.4%) |  | 9 (40.9%) | 6 (20%) |  |
| **The equipment helped in my care at home** |  |  | .4814 |  |  | .7463 |
| Strongly disagree | 0 (0%) | 1 (2.7%) |  | 0 (0%) | 1 (3.3%) |  |
| Disagree | 1 (6.3%) | 7 (18.9%) |  | 4 (18.2%) | 4 (13.3%) |  |
| Neither agree nor disagree | 6 (37.5%) | 17 (45.9%) |  | 8 (36.4%) | 14 (46.7%) |  |
| Agree | 5 (31.3%) | 6 (16.2%) |  | 6 (27.3%) | 5 (16.7%) |  |
| Strongly agree | 4 (25%) | 6 (16.2%) |  | 4 (18.2%) | 6 (20%) |  |
| **I felt comfortable interacting with my care team through the Mayo Clinic Care Plan** |  |  | 0.2798 |  |  | 0.9284 |
| Strongly disagree | 0 (0%) | 0 (0%) |  | 0 (0%) | 0 (0%) |  |
| Disagree | 0 (0%) | 3 (8.3%) |  | 1 (4.5%) | 2 (6.9%) |  |
| Neither agree nor disagree | 1 (6.3%) | 6 (16.7%) |  | 3 (13.6%) | 4 (13.8%) |  |
| Agree | 7 (43.8%) | 17 (47.2%) |  | 11 (50%) | 12 (41.4%) |  |
| Strongly agree | 8 (50%) | 10 (27.8%) |  | 7 (31.8%) | 11 (37.9%) |  |
| **It helped me better understand my condition** |  |  | 0.3560 |  |  | 0.7320 |
| Strongly disagree | 0 (0%) | 1 (2.7%) |  | 1 (4.5%) | 0 (0%) |  |
| Disagree | 0 (0%) | 7 (18.9%) |  | 3 (13.6%) | 4 (13.3%) |  |
| Neither agree nor disagree | 9 (56.3%) | 14 (37.8%) |  | 9 (40.9%) | 13 (43.3%) |  |
| Agree | 4 (25%) | 8 (21.6%) |  | 4 (18.2%) | 8 (26.7%) |  |
| Strongly agree | 3 (18.8%) | 7 (18.9%) |  | 5 (22.7%) | 5 (16.7%) |  |
| **It helped me understand how to care for myself** |  |  | 0.5633 |  |  | 0.5439 |
| Strongly disagree | 0 (0%) | 1 (2.7%) |  | 1 (4.5%) | 0 (0%) |  |
| Disagree | 1 (6.3%) | 8 (21.6%) |  | 3 (13.6%) | 6 (20%) |  |
| Neither agree nor disagree | 8 (50%) | 16 (43.2%) |  | 8 (36.4%) | 15 (50%) |  |
| Agree | 4 (25%) | 5 (13.5%) |  | 5 (22.7%) | 4 (13.3%) |  |
| Strongly agree | 3 (18.8%) | 7 (18.9%) |  | 5 (22.7%) | 5 (16.7%) |  |
| **It helped me understand what I should be tracking throughout my care** |  |  | 0.5658 |  |  | 0.6757 |
| Strongly disagree | 0 (0%) | 1 (2.7%) |  | 1 (4.5%) | 0 (0%) |  |
| Disagree | 0 (0%) | 5 (13.5%) |  | 2 (9.1%) | 3 (10%) |  |
| Neither agree nor disagree | 8 (50%) | 16 (43.2%) |  | 9 (40.9%) | 15 (50%) |  |
| Agree | 5 (31.3%) | 9 (24.3%) |  | 5 (22.7%) | 8 (26.7%) |  |
| Strongly agree | 3 (18.8%) | 6 (16.2%) |  | 5 (22.7%) | 4 (13.3%) |  |
| **It helped me understand what steps I could take to improve my health** |  |  | 0.3201 |  |  | 0.4055 |
| Strongly disagree | 0 (0%) | 1 (2.7%) |  | 1 (4.5%) | 0 (0%) |  |
| Disagree | 0 (0%) | 8 (21.6%) |  | 4 (18.2%) | 4 (13.3%) |  |
| Neither agree nor disagree | 9 (56.3%) | 16 (43.2%) |  | 8 (36.4%) | 17 (56.7%) |  |
| Agree | 4 (25%) | 7 (18.9%) |  | 4 (18.2%) | 6 (20%) |  |
| Strongly agree | 3 (18.8%) | 5 (13.5%) |  | 5 (22.7%) | 3 (10%) |  |
| **It helped me communicate with my care team** |  |  | 0.3308 |  |  | 0.6887 |
| Strongly disagree | 0 (0%) | 2 (5.4%) |  | 1 (4.5%) | 1 (3.3%) |  |
| Disagree | 0 (0%) | 5 (13.5%) |  | 2 (9.1%) | 3 (10%) |  |
| Neither agree nor disagree | 2 (12.5%) | 6 (16.2%) |  | 4 (18.2%) | 3 (10%) |  |
| Agree | 11 (68.8%) | 16 (43.2%) |  | 9 (40.9%) | 18 (60%) |  |
| Strongly agree | 3 (18.8%) | 8 (21.6%) |  | 6 (27.3%) | 5 (16.7%) |  |
| **It helped to inform me when to contact my care team about concerning symptoms** |  |  | 0.7677 |  |  | 0.7107 |
| Strongly disagree | 0 (0%) | 2 (5.4%) |  | 1 (4.5%) | 1 (3.3%) |  |
| Disagree | 2 (12.5%) | 7 (18.9%) |  | 3 (13.6%) | 6 (20%) |  |
| Neither agree nor disagree | 6 (37.5%) | 9 (24.3%) |  | 5 (22.7%) | 10 (33.3%) |  |
| Agree | 5 (31.3%) | 12 (32.4%) |  | 7 (31.8%) | 9 (30%) |  |
| Strongly agree | 3 (18.8%) | 7 (18.9%) |  | 6 (27.3%) | 4 (13.3%) |  |
| **Please rate your level of agreement with the following statements about the education provided while using the Mayo Clinic Care Plan.** |  |  |  |  |  |  |
| **The educational materials were useful to me** |  |  | 0.4333 |  |  | 0.5595 |
| Strongly disagree | 0 (0%) | 2 (5.7%) |  | 1 (4.5%) | 1 (3.6%) |  |
| Disagree | 1 (6.3%) | 5 (14.3%) |  | 3 (13.6%) | 3 (10.7%) |  |
| Neither agree nor disagree | 6 (37.5%) | 6 (17.1%) |  | 5 (22.7%) | 7 (25%) |  |
| Agree | 7 (43.8%) | 15 (42.9%) |  | 7 (31.8%) | 14 (50%) |  |
| Strongly agree | 2 (12.5%) | 7 (20%) |  | 6 (27.3%) | 3 (10.7%) |  |
| **The information was easy to understand** |  |  | 0.8373 |  |  | 0.6221 |
| Strongly disagree | 0 (0%) | 1 (2.9%) |  | 0 (0%) | 1 (3.6%) |  |
| Disagree |  |  |  | 0 (0%) | 0 (0%) |  |
| Neither agree nor disagree | 3 (18.8%) | 8 (22.9%) |  | 5 (22.7%) | 5 (17.9%) |  |
| Agree | 8 (50%) | 18 (51.4%) |  | 10 (45.5%) | 16 (57.1%) |  |
| Strongly agree | 5 (31.3%) | 8 (22.9%) |  | 7 (31.8%) | 6 (21.4%) |  |
| **I was comfortable with how often I received educational materials** |  |  | 0.6569 |  |  | 0.1507 |
| Strongly disagree | 0 (0%) | 2 (5.7%) |  | 1 (4.5%) | 1 (3.6%) |  |
| Disagree | 1 (6.3%) | 2 (5.7%) |  | 1 (4.5%) | 1 (3.6%) |  |
| Neither agree nor disagree | 2 (12.5%) | 9 (25.7%) |  | 7 (31.8%) | 4 (14.3%) |  |
| Agree | 10 (62.5%) | 16 (45.7%) |  | 7 (31.8%) | 19 (67.9%) |  |
| Strongly agree | 3 (18.8%) | 6 (17.1%) |  | 6 (27.3%) | 3 (10.7%) |  |
| **I was able to find the educational materials when I needed them** |  |  | 0.7865 |  |  | 0.1294 |
| Strongly disagree | 1 (6.3%) | 2 (5.7%) |  | 0 (0%) | 3 (10.7%) |  |
| Disagree | 0 (0%) | 2 (5.7%) |  | 1 (4.5%) | 1 (3.6%) |  |
| Neither agree nor disagree | 3 (18.8%) | 10 (28.6%) |  | 7 (31.8%) | 6 (21.4%) |  |
| Agree | 9 (56.3%) | 16 (45.7%) |  | 8 (36.4%) | 16 (57.1%) |  |
| Strongly agree | 3 (18.8%) | 5 (14.3%) |  | 6 (27.3%) | 2 (7.1%) |  |
| **The educational materials matched my personal needs** |  |  | 0.7934 |  |  | 0.2971 |
| Strongly disagree | 0 (0%) | 1 (2.9%) |  | 0 (0%) | 1 (3.6%) |  |
| Disagree | 1 (6.3%) | 4 (11.4%) |  | 2 (9.1%) | 3 (10.7%) |  |
| Neither agree nor disagree | 8 (50%) | 12 (34.3%) |  | 8 (36.4%) | 12 (42.9%) |  |
| Agree | 5 (31.3%) | 14 (40%) |  | 7 (31.8%) | 11 (39.3%) |  |
| Strongly agree | 2 (12.5%) | 4 (11.4%) |  | 5 (22.7%) | 1 (3.6%) |  |
| **The educational information from the Mayo Clinic Care Plan matched the information received from my Mayo Clinic Care Team** |  |  | 0.6313 |  |  | 0.7024 |
| Strongly disagree | 0 (0%) | 1 (2.9%) |  | 0 (0%) | 1 (3.6%) |  |
| Disagree | 0 (0%) | 0 (0%) |  | 0 (0%) | 0 (0%) |  |
| Neither agree nor disagree | 4 (25%) | 14 (40%) |  | 8 (36.4%) | 10 (35.7%) |  |
| Agree | 8 (50%) | 13 (37.1%) |  | 8 (36.4%) | 12 (42.9%) |  |
| Strongly agree | 4 (25%) | 7 (20%) |  | 6 (27.3%) | 5 (17.9%) |  |
| **Please rate your level of agreement with the following statements.** |  |  |  |  |  |  |
| **I would recommend the Mayo Clinic Care Plan to others with similar health condition(s)** |  |  | 0.7896 |  |  | 0.5359 |
| Strongly disagree | 0 (0%) | 1 (2.9%) |  | 1 (4.5%) | 0 (0%) |  |
| Disagree | 0 (0%) | 2 (5.7%) |  | 0 (0%) | 2 (7.4%) |  |
| Neither agree nor disagree | 3 (20%) | 5 (14.3%) |  | 3 (13.6%) | 5 (18.5%) |  |
| Agree | 8 (53.3%) | 16 (45.7%) |  | 11 (50%) | 12 (44.4%) |  |
| Strongly agree | 4 (26.7%) | 11 (31.4%) |  | 7 (31.8%) | 8 (29.6%) |  |
| **Overall, I am satisfied with the Mayo Clinic Care Plan** |  |  | 0.3848 |  |  | 0.2457 |
| Strongly disagree | 0 (0%) | 1 (2.9%) |  | 1 (4.5%) | 0 (0%) |  |
| Disagree | 0 (0%) | 4 (11.4%) |  | 0 (0%) | 4 (14.8%) |  |
| Neither agree nor disagree | 1 (6.7%) | 6 (17.1%) |  | 4 (18.2%) | 3 (11.1%) |  |
| Agree | 10 (66.7%) | 15 (42.9%) |  | 10 (45.5%) | 14 (51.9%) |  |
| Strongly agree | 4 (26.7%) | 9 (25.7%) |  | 7 (31.8%) | 6 (22.2%) |  |
